# Impairments of functional exercise capacity, muscle strength, balance and kinesiophobia in patients with chronic kidney disease: a cross-sectional study

**DOI:** 10.1186/s12882-023-03448-z

**Published:** 2024-01-11

**Authors:** Nihan Katayıfçı, İrem Hüzmeli, Döndü İriş, Faruk Hilmi Turgut

**Affiliations:** 1 Faculty of Health Sciences, Department of Physiotherapy and Rehabilitation, Hatay Mustafa Kemal University, Hatay, Turkey; 2 Institute of Health Sciences, Department of Physiotherapy and Rehabilitation, Hatay Mustafa Kemal University, Hatay, Turkey; 3 Tayfur Ata Sokmen Faculty of Medicine, Department of Nephrology, Hatay Mustafa Kemal University, Hatay, Turkey

**Keywords:** Chronic Kidney Disease, Exercise capacity, Muscle strength, Balance, Kinesiophobia

## Abstract

**Background:**

Muscle weakness, balance, and functional capacity are affected in patients with chronic kidney disease (CKD) in dialysis. However, studies about kinesiophobia, peripheral and respiratory muscle strength, balance, exercise capacity, fatigue, and physical activity level in patients with CKD 3–4 are limited. The study aimed to compare the functional exercise capacity, peripheral and respiratory muscle strength, pulmonary function, balance, kinesiophobia, physical activity, fatigue, and dyspnea between patients with CKD 3–4 and controls.

**Methods:**

This cross-sectional study included 43 patients and 45 controls. Functional exercise capacity [6-Minute Walking Test (6MWT)], peripheral and respiratory muscle strength, pulmonary function, dyspnea, fatigue, physical activity, balance [Berg Balance Scale (BBS)], and kinesiophobia were evaluated.

**Results:**

Demographic characteristics were similar in patients [53(50–57) y, 26 M/17F] and controls [51(4.506-55) y, 33 M/12F] (*p* > 0.05). The 6MWT, respiratory and peripheral muscle strength, pulmonary function, physical activity, and BBS were significantly lower, and the level of dyspnea and kinesiophobia were higher in patients compared with controls (*p* < 0.05).

**Conclusions:**

Patients had impaired functional exercise capacity, upper and lower extremity muscle strength, respiratory muscle strength, pulmonary function, and balance, increased perception of dyspnea and kinesiophobia, and reduced physical activity level compared with controls. Patients should be directed to cardiopulmonary rehabilitation programs.

## Introduction

Chronic kidney disease (CKD) is a global health problem characterized by irreversible kidney damage directly affecting normal kidney function, impacting approximately 700 million people worldwide, and associated with high economic costs [1]. CKD patients are at high risk of cardiovascular mortality, which is associated with age-related decline in kidney function, hypertension, diabetes, and obesity [2]. Exercise intolerance is one of the most critical risk factors for cardiac mortality. In addition, exercise capacity decreases both in patients with CKD stage 2–5 and end-stage renal disease (in hemodialysis) [3, 4]. Moreover, anemia, volume overload, and muscle wasting trigger exercise intolerance [5].

Chronic kidney disease impacts several systems, including the respiratory and musculoskeletal systems [6–9]. Loss of muscle proteins lead to muscle atrophy and decrease muscle strength [10]. Factors that cause muscle protein loss include metabolic acidosis, inflammation, insulin resistance, malnutrition, changes in hormones, oxidative stress and physical inactivity [10]. Decrease in hand-grip and quadriceps femoris muscle strength have been shown in patients with CKD 3b-5 not started dialysis and hemodialysis [9, 11]. However, there has been no information about involvement in proximal upper extremity. Shorten myogenic fibers cause higher muscle tension in diaphragm due to renal dysfunction. Weakness in respiratory muscle was seen in patients with CKD stage 5 non-dialysis [12] and on dialysis [8, 12]. However, effect of CKD on respiratory muscle strength in patients with early stages (3–4) is not known. Changes in phosphorus metabolism and hormones, oxidative stress, elevation of proinflammatory cytokines, acid-base imbalance, fluid overload lead to development of respiratory diseases in patients with CKD [13]. Therefore, respiratory system assessment is very crucial in patients with CKD. Fatigue has a direct negative relationship with physical activity in patients after kidney transplantation and indirectly affects physical activity through mediating effects of physical self-efficacy and kinesiophobia [14, 15]. However, presence of kinesiophobia is not known in CKD stages 3–4. Postural stability is impaired in patients with CKD 3b-5, and poor postural stability, which decreases with reduced renal function, is associated with decreased physical and cognitive function [9]. Muscle weakness, kinesiophobia, and functional capacity are affected in CKD patients with dialysis or transplantation [8, 14]. However, in the literature, results involving peripheral and respiratory muscle strength, balance, exercise capacity, fatigue, physical activity level kinesiophobia, and other related factors in patients with CKD 3–4 are scarce. Although CKD is irreversible, slowing the progression in early stages might decrease mortality and comorbidities [16]. It is essential to identify impairments with a wide range of evaluation. Therefore, the current study aimed to compare functional exercise capacity, peripheral and respiratory muscle strength, pulmonary function, balance, kinesiophobia, physical activity, fatigue, and dyspnea between patients with CKD 3–4 and controls.

## Methods

### Patients

This cross-sectional study included 45 healthy controls and 43 patients referred to the cardiopulmonary rehabilitation department between July 2021 and February 2022. The study included patients diagnosed with CKD at stage 3–4, aged ≥ 18 years, and clinically stable for at least 4 weeks. Patients with orthopedic, neurological, or pulmonary diseases, uncontrolled cardiovascular disease, malignancies, severe anemia, and diabetic polyneuropathy were excluded. The Ethics Committee of the Hatay Mustafa Kemal University approved the study (No: 2021/34). Informed consent was obtained from patients and controls in the study following the Declaration of Helsinki principles.

### Assessments

Demographic and clinical characteristics (blood biochemistry data) were recorded. Spontaneous gait speed was evaluated using a 4-meter course. Participants walked at their usual speed, and the mean duration of two trials was recorded as spontaneous gait speed. The patients were evaluated over 2 consecutive days. On the first day 6-minute walk tests (6MWT), physical activity level, dyspnea and fatigue perception were performed; pulmonary function, peripheral and respiratory muscle strength, balance, kinesiophobia measurements were performed the other day. The 6MWT was conducted in the morning, with the second test administered after a minimum 30-minute interval. This break allowed patients to recuperate from fatigue and dyspnea, ensuring they returned to resting levels before the subsequent evaluation.

Functional exercise capacity was evaluated with the 6-Minute Walking Test (6-MWT) according to the American Thoracic Society (ATS) criteria [17]. Participants were instructed to walk at their average speed in a 30-m unobstructed corridor. The test was performed twice with a rest period of 30 min. The best distance was used for the analysis. For comparison, reference values were used [18]. The 6- Minute Walking Work (6-MWw) was calculated as the product of the most significant 6-MWT distance (in kilometers) and weight (in kilograms) [19].

A hand-held dynamometer (JTECH Power Track Commander, Baltimore, MD, USA) was used to evaluate shoulder abductors and quadriceps femoris muscle strength. According to the reference values, the percentage of the predicted value was calculated [20]. Hand-grip strength was assessed with a Jamar analog hand dynamometer (PowerTrack II, JTECH Medical, Midvale, Utah, USA) [21]. Measurements were performed three times, and the highest value was used for comparison.

Pulmonary function was evaluated with a portable spirometer (Spirobank MIR, Rome, Italy). Forced vital capacity (FVC), forced expiratory volume in one second (FEV_1_), peak expiratory flow (PEF), and forced expiratory flow from 25 to 75% (FEF_25–75%_) are expressed as percentages of the predicted values [22].

A mouth pressure device (Micro Medical MicroRPM, England) was used to assess maximal inspiratory pressure (MIP) and maximal expiratory pressure (MEP) according to ATS/ERS guidelines [23]. Reference values were used for comparison [24]. Respiratory muscle weakness is considered MIP and MEP < 80% of the predicted values [23].

Balance was evaluated with the Functional Reach Test (FRT) and Berg’s balance scale (BBS). The FRT measures the distance in centimeters between the length of an outstretched arm in a maximal forward reach without losing balance [25]. The BBS consists of 14 balance-related tests scoring zero to four. The maximum score is 56. Higher scores show better balance [26].

The kinesiophobia was assessed with the Tampa Scale of Kinesiophobia (TSK). The TSK consists of seventeen items, each with a 4-point response scale ranging from “strongly disagree” to “strongly agree”. The total score ranges between 17 and 68. Higher scores indicate a higher level of kinesiophobia. The cut-off point for a high level of kinesiophobia is considered as above 37 [27].

The Fatigue Severity Scale (FSS) was used to evaluate fatigue. The scale includes nine items. Each item is scored 0 (strong disagreement) to 7 (strong agreement). The total score ranges from 0 to 63. Scores above 36 indicate severe fatigue [28].

The Modified Medical Research Council (MMRC) dyspnea scale was used to evaluate dyspnea. Dyspnea levels, which include the statements that best describe the dyspnea level, were graded between 0 and 4 [29].

The physical activity level was evaluated with the International Physical Activity Questionnaire (IPAQ) short-form. The questionnaire contains information about walking time, moderate and vigorous-intensity activity, and sitting duration. Each category of physical activity was calculated by multiplying the recorded minutes and frequency per week within every activity by a metabolic equivalent (MET) energy expenditure calculation. The scores were categorized as inactive (< 600 MET-min/week), minimally active (600–3000 MET-min/week), and sufficiently active (> 3000 MET-min/week) [30].

### Statistical analysis

SPSS 20.0 statistical analysis program was used (Armonk, NY: IBM Corp). Based on the results of a prior study [31], the sample size (G*Power 3.0.10 system, Franz Faul, Universität Kiel, Germany) was estimated to be at least 26 individuals for each group to detect an α value of 0.05, an effect size of 0.95, and a power of 95%. The Shapiro-Wilk and Kolmogorov–Smirnov tests were used to assess the normality of the data. Data are expressed as mean (± standard deviation), mean difference, and 95% CI for distributed data, and Student’s t-test was used to compare. The Mann–Whitney U test was used to compare undistributed data expressed as the median (IQR). The Chi-square test was used for the comparison of the nominal data. Pearson’s and Spearman’s rank correlation coefficients were used to calculate correlations between BBS, TKS, demographic, and clinical factors. A *p*-value < 0.05 was considered statistically significant.

## Results

**Fig. 1:**
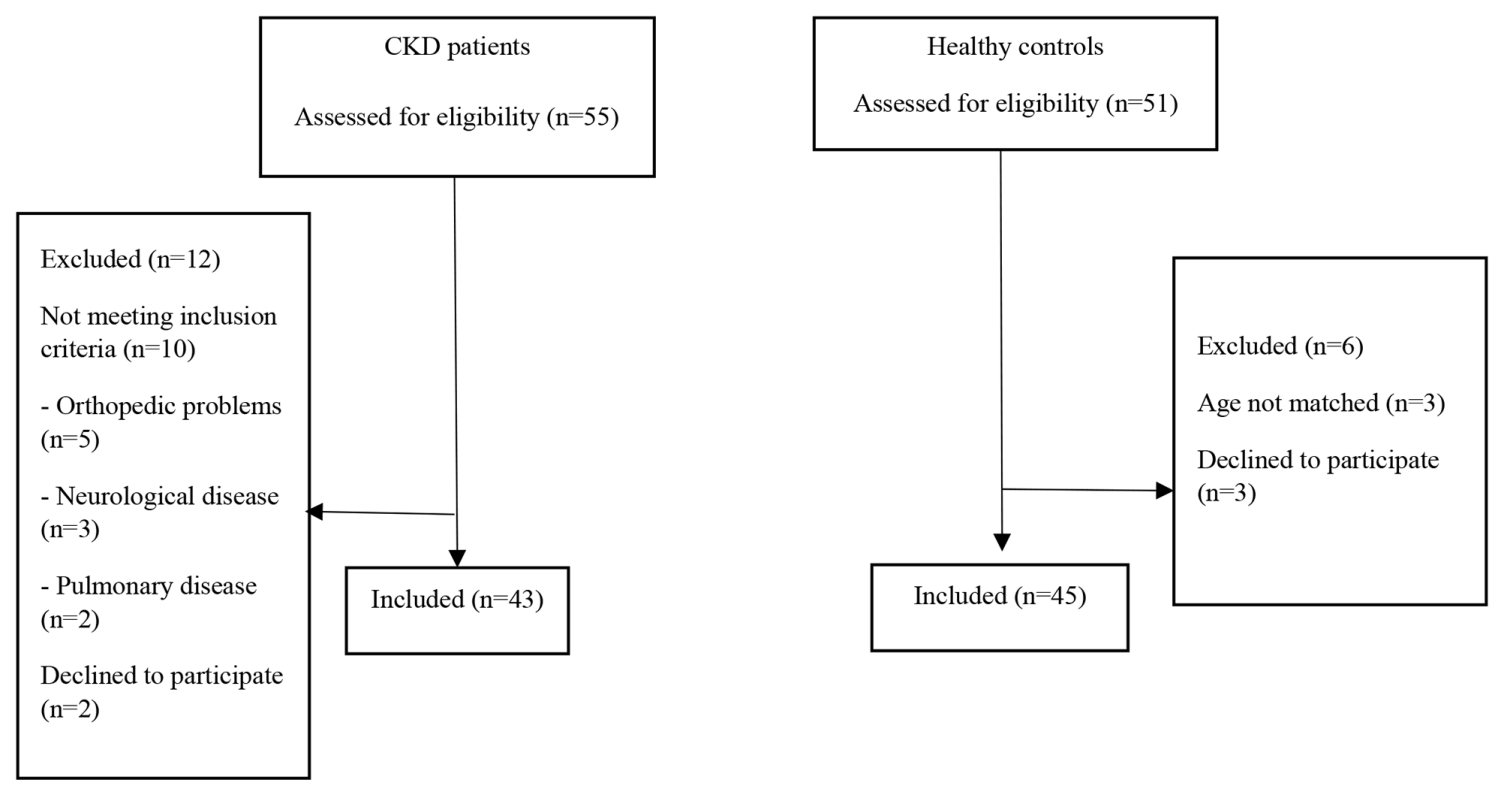
Flow diagram of the patients with CKD and controls

Forty-three patients with CKD and 45 controls were enrolled in the present study (Fig. 1). Demographic and clinical characteristics of the patients and controls were similar (*p* > 0.05) (Table 1). The CKD stages of the patients were stage III (n = 27, 62.8%) and stage IV (n = 16, 37.2%).

**Table 1:** Demographic characteristics of patients with CKD and controls

Variables	Patients with CKD Mean ± SD Median (IQR)	Control subjects Mean ± SD Median (IQR)	Mean difference 95% CI	*p*
Age (years)	53(50–57)	51(46.50–55)		0.165
Sex (male/female)	26/60.5%; 17/39.5%	33/73.3%; 12/26.7%		0.199
Weight, kg	84(78.79-94)	80(74.50–85)		0.102
Height, cm	170(161–175)	167(165–177)		0.616
BMI, kg/m^2^	29.17 ± 3.22	27.80 ± 3.22	-1.37(-3.22-0.47)	0.138
Smoking (pack/year)	0(0-17.50)	0(0-17.50)		0.967
Smoking (current/ex/non-smoker), n (%)	8/18.6%;10/23.3%;25/58.1%	16/35.6%;7/15.6%;22/48.9%		0.188
eGFR (mL/min/1.73^2)^	37.86(29.71–53.59)			
Hemoglobin (g/L)	12.71 ± 1.90			

BMI: body mass index; CKD: chronic kidney disease; eGFR: estimated glomerular filtration rate; CI: confidence interval. *p˂0.05

**Fig. 2:**
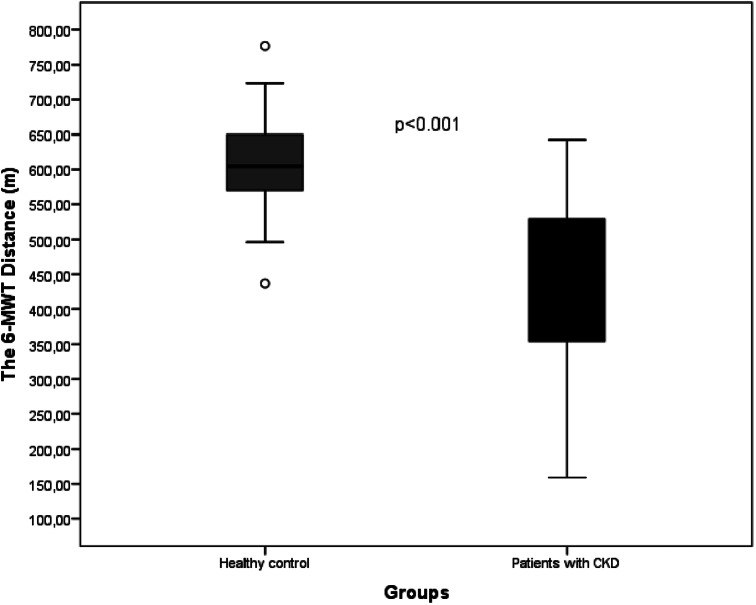
Comparison of 6-MWT distance in patients with CKD and controls

The comparison of the 6-MWT parameters between patients with CKD and controls is shown in Table 2. The 6-MWT distance (*p* < 0.001) (Figs. 2), 6-MWT% (*p* < 0.001), and 6MWw (*p* < 0.001) were significantly lower in patients compared with controls (Table 2). Twenty-nine (67.4%) patients had values less than 80% of the predicted 6-MWT.

**Table 2:** Comparison of 6-MWT parameters in patients with CKD and controls

6-MWT parameters	Patients with CKD Mean ± SD Median (IQR)	Control subjects Mean ± SD Median (IQR)	Mean difference %95 CI	*p*
6-MWT distance, m	444.60(354–538)	604.80(566.40-650.50)		< 0.001*
6-MWT distance, % predicted	70.19 ± 17.84	94.45 ± 13.29	24.25(17.60–30.90)	< 0.001*
6MWw, kg/m	35654.40(26923.20-45896.40)	50,310(42687-54518.10)		< 0.001*
Heart rate, beats/min (resting)	76.62 ± 13.02	82.06 ± 11.84	5.43(0.16–10.70)	0.043*
Peak heart rate, beats/min	103.25 ± 18.48	108.51 ± 24.24	5.25[(-3.91)-14.42]	0.258
Maximum heart rate,%	61.91 ± 10.95	64.41 ± 15.14	2.50(-3.11-8.12)	0.378
Systolic blood pressure, mmHg (resting)	130(120–140)	120(110-122.50)		0.005*
∆ Systolic blood pressure, mmHg	10(-10-20)	10(3–30)		0.023*
Diastolic blood pressure, mmHg (resting)	80(70–80)	75(70–80)		0.469
∆ Diastolic blood pressure, mmHg	0(-10-10)	10(0–10)		0.002*
SpO_2_, % (resting)	98(97–98)	98(97–98)		0.664
∆ SpO_2_, %	-1(-2-0)	0(-3-1)		0.747
Breathing frequency, breaths/min (resting)	22(20–24)	21(19.50–24)		0.898
∆ Breathing frequency, breaths/min	4(2–8)	5(4–8)		0.215
Dyspnea, 0–10 (resting)	0(0–0)	0(0–0)		1.000
∆ Dyspnea, 0–10	0(0–3)	0(0-1.50)		0.159
Fatigue, 0–10 (resting)	0(0–0)	0(0–0)		0.328
∆ Fatigue, 0–10	1(0–3)	0(0–3)		0.467

6-MWT: 6-minute walk test; SpO2: Oxygen saturation; 6MWw: 6-minute walk distance x body weight; CI: confidence interval. *p < 0.05

**Fig. 3:**
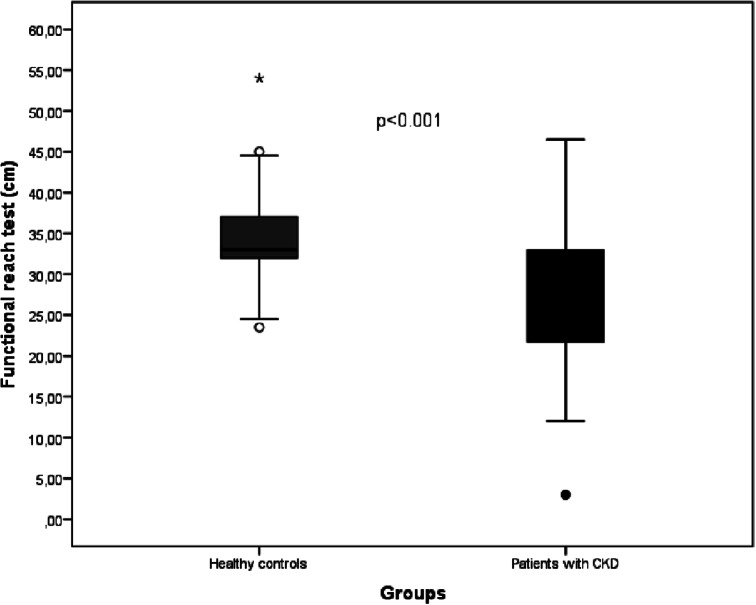
Comparison of FRT in patients with CKD and controls

The comparison of pulmonary function, muscle strength, dyspnea, kinesiophobia, fatigue, balance, and physical activity level scores in patients with CKD and controls is presented in Table 3. Predicted FEV1, FVC, PEF, FEF25-75%, measured and predicted MIP, MEP, measured and predicted shoulder abductors and quadriceps femoris, hand grip strength, BBS score, FRT (Fig. 3), IPAQ total, and vigorous, moderate, spontaneous gait speed were significantly lower in patients compared with controls (*p* < 0.05, Table 3). The MMRC dyspnea score, modified Borg score, TSK, and IPAQ sitting duration were higher in patients than in controls (*p* < 0.05, Table 3). Eighteen (41.9%) patients had less than 80% of predicted MIP, and 29 (67.4%) patients had less than 80% of predicted MEP. Thirteen patients (30.2%) had less than 75% of predicted FVC, 19 patients (44.2%) had less than 75% of predicted FEV_1_, 13 patients (30.2%) had less than 75% of predicted FEV_1_/FVC, 36 patients (83.7%) had less than 75% of predicted PEF, and 30 patients (69.8%) had less than 75% of predicted FEF_25–75%_. Forty-one (95.3%) patients had less than 80% of the predicted quadriceps femoris strength, and 33 (76.7%) had less than 80% of the predicted shoulder abductors strength. Twenty-five (58.1%) patients were inactive, 14 (32.6%) were minimally active, and 4 (9.3%) were sufficiently active, while 16 (38.1%) of the controls were inactive, 11 (26.2%) were minimally active, and 15 (35.7%) were sufficiently active (*p* = 0.013). Twenty-seven (62.8%) patients reported severe fatigue, and 23 (53.5%) patients had a high level of kinesiophobia.

**Table 3:** Comparison of pulmonary function, respiratory and peripheral MS, dyspnea, kinesiophobia, fatigue, balance, PA level scores in patients with CKD and controls

Variables	CKD patients Mean ± SD Median (IQR)	Control Mean ± SD Median (IQR)	Mean difference %95 CI	*p*
FEV_1_ (%)	78.48 ± 18.13	91.04 ± 15.06	12.55(5.50–19.60)	0.001*
FVC (%)	83.30 ± 15.32	93.31 ± 14.26	10(3.73–16.27)	0.002*
FEV_1_/FVC	78.90(68.10–88.80)	81.90(78.05–87.15)		0.339
PEF (%)	52.32 ± 21.28	72.95 ± 23.06	20.62(11.08–30.17)	< 0.001*
FEF_%25−75_ (%)	62(49–81)	83(67-110.50)		< 0.001*
MIP (cmH_2_O)	80.16 ± 31.55	105.68 ± 31.96	25.52(12.06–38.99)	< 0.001*
%MIP	88.37 ± 31.17	113.23 ± 29.87	24.86(11.92–37.79)	< 0.001*
MEP (cmH_2_O)	84(65–109)	120(88.50–154)		< 0.001*
%MEP	70.44(62.73–89.22)	101.42(76.85–131.10)		< 0.001*
Quadriceps femoris, (Left), N	147(107–235)	185(163.50–220)		0.003*
Quadriceps femoris, (Right)	147(116–228)	184(170-221.50)		0.005*
%Quadriceps femoris, (ND)	35.09(26.96–53.30)	43.87(37.01–50.44)		0.012*
Shoulder abductors (Left), N	103(81.40–149)	140(113–176)		0.002*
Shoulder abductors (Right), N	110(79.20–145)	154(116–193)		< 0.001*
%Shoulder abductors (ND)	61.63(44.54–78.10)	73.84(64.53–88.69)		0.007*
Handgrip, (Left), P	67.72 ± 25.47	79.08 ± 21.76	11.36(1.34–21.39)	0.027*
Handgrip, (Right), P	69.32 ± 26.57	83.82 ± 21.98	14.49(4.17–24.81)	0.006*
MMRC dyspnea scale score, 0–4	1(0–1)	0(0–0)		< 0.001*
Modified Borg Scale score, 0–10 (Activity)	2(1–4)	0-(0–2)		< 0.001*
TSK score (17–68)	37.48 ± 7.98	32.17 ± 8.73	-5.31[(-8.86)-(-1.75)]	0.004*
FSS score (0–63)	26(6–56)	14(5.50–30)		0.082
BBS score (0–56)	51(46–56)	56(56–56)		< 0.001*
FRT (cm)	26.50(21.50–33)	33(32-37.25)		< 0.001*
IPAQ (MET-min/week)				
Total	462(99–924)	1792.50(405.75-3692.25)		0.001*
Walking	346(99–693)	478.50(132–1386)		0.120
Moderate	0(0–0)	0(0-1500)		0.010*
Vigorous	0(0–0)	0(0-1080)		0.006*
Sitting (min/day)	360(300–540)	240(180-457.50)		0.006*
Spontaneous gait speed (m/s)	1.03 ± 0.32	1.34 ± 0.34	0.30(0.16–0.44)	< 0.001*

MS: muscle strength; PA: physical activity; FEV_1_: Forced expiratory volume in one second; FVC: Forced vital capacity; PEF: Peak expiratory flow, FEF_25 − 75%_: Forced expiratory flow from 25–75%; MIP: Maximal inspiratory pressure; MEP: Maximal expiratory pressure; ND: non-dominant; MMRC: Modified Medical Research Council Dyspnea Scale; TSK: Tampa Scale of Kinesiophobia; FSS: Fatigue Severity Scale; BBS: Berg Balance Scale; FRT: Functional reach test; IPAQ, International Physical Activity Questionnaire. *p < 0.05

Table 4 shows a correlation between BBS, TKS, and demographic and clinical characteristics of patients with CKD. The BBS was significantly correlated with age, weight, BMI, quadriceps femoris muscle strength, 6-MWT distance, FRT, and spontaneous gait speed. The TKS was significantly related to MEP, quadriceps femoris muscle strength, 6-MWT distance, and fatigue (*p* < 0.05, Table 4). In the multiple regression analysis conducted, 54.8% of the variance in the BBS was explained by weight (β= -0.196, *p* = 0.012) and FRT (β = 0.210, *p* = 0.033), and 38.1% of the TKS was explained by MEP (β= -0.099, *p* = 0.005) and FSS (β= = -0.109, *p* = 0.037).

**Table 4:** Correlations among BBS and TKS with demographic and clinic characteristics of patients with CKD

	BBS		TKS	
Characteristics	r value	*p* value	r value	*p* value
Age, years	**-0.532**	< 0.001*	0.148	0.345
Male/female, n	-0.081	0.604	-0.231	0.137
Weight, kg	**-0.384**	0.011*	-0.226	0.146
Height, cm	-0.054	0.730	-0.096	0.542
BMI, kg/m2	**-0.352**	0.021*	-0.212	0.171
Smoking (pack/year)	-0.103	0.509	-0.047	0.765
FEV_1_ (%)	0.022	0.890	-0.087	0.580
FVC (%)	0.006	0.970	-0.077	0.623
FEV_1_/FVC	0.196	0.209	-0.069	0.658
PEF (%)	0.053	0.735	-0.075	0.631
FEF_%25−75_ (%)	0.107	0.497	-0.144	0.358
MIP (cmH_2_O)	0.109	0.487	-0.224	0.149
MEP (cmH_2_O)	-0.002	0.991	**-0.438**	0.003*
Quadriceps femoris, (Left), N	**0.333**	0.029*	**-0.300**	0.044*
Quadriceps femoris, (Right)	0.297	0.053	-0.122	0.436
%Quadriceps femoris, (ND)	**0.418**	0.005*	-0.133	0.395
Shoulder abductors (Left), N	0.144	0.358	-0.167	0.285
Shoulder abductors (Right), N	0.091	0.560	-0.073	0.640
%Shoulder abductors (ND)	0.199	0.200	0.050	0.751
Handgrip, (Left), P	0.196	0.207	-0.234	0.131
Handgrip, (Right), P	0.123	0.433	-0.251	0.105
MMRC dyspnea scale score, 0–4	0.181	0.245	-0.148	0.342
Modified Borg Scale score, 0–10 (Activity)	-0.124	0.430	-0.124	0.430
6-MWT distance, m	**0.499**	0.001*	**-0.353**	0.020*
TSK score (17–68)	-0.164	0.292	-	-
FRT (cm)	**0.512**	< 0.001*	-0.021	0.896
Spontaneous gait speed (m/s)	**0.514**	< 0.001*	-0.137	0.379
FSS score (0–63)	-0.189	0.226	**0.446**	0.003*
IPAQ (MET-min/week)				
Total	0.093	0.552	-0.038	0.811
Walking	0.119	0.447	0.013	0.936
Moderate	0.042	0.791	-0.058	0.714
Vigorous	-0.051	0.746	-0.170	0.275
Sitting (min/day)	-0.284	0.065	0.074	0.638
BBS score (0–56)	-	**-**	-0.164	0.292

BMI: Body mass index; FVC: Forced vital capacity; FEV_1_: Forced expiratory volume in one second; PEF: Peak expiratory flow, FEF_25 − 75%_: Forced expiratory flow from 25–75%, MIP: Maximal inspiratory pressure; MEP: Maximal expiratory pressure; ND: non-dominant; MMRC: Modified Medical Research Council Dyspnea Scale; 6-MWT: 6-minute walk test; TSK: Tampa Scale of Kinesiophobia; FRT: Functional reach test; FSS: Fatigue Severity Scale; IPAQ, International Physical Activity Questionnaire; BBS: Berg Balance Scale. *p < 0.05. r: Pearson/spearman correlation coefficients

## Discussion

This study aimed to compare the functional exercise capacity, peripheral, and respiratory muscle strength, pulmonary function, balance, kinesiophobia, physical activity, fatigue, and dyspnea between patients with CKD 3–4 and controls and the most important findings of the present study were: (1) functional exercise capacity and upper and lower extremity muscle strength, balance, respiratory muscle strength, and pulmonary functions were lower in the patient group compared to controls; (2) dyspnea and kinesiophobia levels were higher in the patient group compared to controls; (3) physical activity level was lower in the patient group compared to controls; and (4) weight was determinant of (54.8%) balance and expiratory MS and fatigue were determinants of (38.1%) kinesiophobia in patients with CKD.

In previous studies, exercise intolerance was shown in patients with CKD [3, 7, 31]. A previous study reported that patients with CKD at stage 2–5 whose exercise capacity decreased over 5 years had low physical activity levels [7]. Another study showed that patients with CKD on hemodialysis had lower 6-MWT than healthy controls. Additionally, they stated that patients achieved a 101.5 m shorter distance compared to controls [31]. In addition to exercise capacity, a systematic review showed that gait speed was lower in patients with CKD, and it decreased with increasing CKD severity [32]. Moreover, gait speed was found to be an independent predictor of the 6-MWT [31]. In accordance with the previous literature, in the current study, 6-MWT distance and gait speed were lower in patients than in controls. Studies investigating the effects of interventions improving exercise capacity and gait speed in patients with CKD are needed.

A study showed that postural balance performance was lower in faller end-stage renal disease (dialysis) patients living with a kidney transplant compared to non-fallers [8]. Another study showed that balance was impaired in CKD patients with stage 3b-5. Additionally, they stated that GFR was negatively correlated with the functional reach test [9]. In the current study, BBS and FRT were lower in patients at stage 3–4 CKD than in healthy controls. Only weight was negatively correlated with the balance. Other factors that may affect balance should be investigated in patients with CKD in early stages.

Hemodialysis or kidney transplant is known to adversely affect muscle strength [11, 33]. Ankle dorsiflexion and quadriceps femoris muscle strength were lower in end-stage renal disease patients living with a kidney transplant [33]. Another study showed that quadriceps femoris muscle and hand-grip strength were lower in hemodialysis patients than in nondialysis patients [11]. Reductions of ~10% in quadriceps femoris and ~16% in hand-grip strength were found in patients with CKD 3b-5 not started dialysis [9]. In the current study, in addition to the quadriceps femoris muscle and hand-grip strength, shoulder abductor muscle strength was also weakened in patients with CKD 3–4. The present study firstly showed a decline in proximal upper extremity muscle strength. Proximal upper extremity assessment should be considered in cardiac rehabilitation programs in patients.

Pathological changes in the diaphragm result in a decline of respiratory muscle strength [34]. Weakened respiratory muscle strength (MIP and MEP) was shown in patients with CKD at stage 5 non-dialysis [12] and on dialysis [8, 12]. In the current study, inspiratory and expiratory MS were lower in patients with CKD at stage 3–4. Additionally, 41.9% of patients had less than 80% of the predicted MIP and 67.4% of patients had less than 80% of the predicted MEP. A decrease in respiratory muscle strength was firstly shown in early stage (stage 3–4) in patients with CKD. Therefore, it is very crucial to identify respiratory muscle weakness in early stages and include patients respiratory muscle training programs to overcome respiratory muscle weakness. Additionally, MEP was inversely correlated with kinesiophobia. Factors that might affect respiratory muscle strength should be investigated in future studies.

Kidney disease directly or indirectly affects the mechanical properties and ventilation of the lungs [35]. Impact of lung diseases might impair kidney function, particularly when coupled with other comorbidities. Furthermore, lung diseases independently correlate with elevated mortality rates among patients with CKD [13]. Therefore, the relationship between lung and kidney function is crucial. Both obstructive and restrictive pulmonary abnormality patterns were seen in patients with CKD. The study stated that 10%, 16% of the patients with CKD stage 1–4 had restrictive and obstructive lung function abnormality, respectively [35]. In the current study, 30.2%, 30.2%, and 69.8% of the patients, respectively, had restrictive pulmonary function abnormality, obstructive pulmonary function abnormality, and small airway obstruction. The difference might be due to not including stages 1–2 in the current study. It is known that as the renal function decreases, it has been seen an increase in prevalence of lung disease [13]. Albuminuria, smoking status, and older age were correlated with obstructive and restrictive respiratory pulmonary abnormality patterns and lower eGFR was associated with obstructive patterns [35]. Moreover, mechanism such as changes in phosphorus metabolism and hormones, oxidative stress, elevation of proinflammatory cytokines, acid-base imbalance, fluid overload, contribute respiratory problems in patients with CKD [13]. Although patients with pulmonary disease were not included in the current study, factors that are mentioned above might cause respiratory problems. Factors affecting lung function and exposure level at different stages should be investigated in patients with CKD. It was stated that routine clinical practice in the management of patients with CKD typically does not include the assessment of respiratory function [36]. Importance should be given to respiratory function assessment for the management of CKD.

Fatigue, the most common symptom of patients with CKD, affects 70% of patients. Patients experience fatigue in early stages of CKD (2–3). In addition, the prevalence elevates with CKD stages [37]. In a review it was stated that severe fatigue was seen in 25% of patients with CKD (all stages) [38]. Lactic acidosis, chronic metabolic acidosis, and depression contribute to fatigue [38]. In the current study, 62.8% of the patients reported severe fatigue. Additionally, fatigue was correlated with kinesiophobia. Peripheral muscle impairment, exercise intolerance, and physical inactivity may lead to fatigue. Early rehabilitation programs should be developed to overcome fatigue.

Dyspnea is a common multifactorial symptom in patients with CKD [39]. Salerno et al. stated that contributors to dyspnea were less understood [39]. In the present study, MMRC dyspnea and modified Borg scores were higher in patients with CKD than in controls. More attention should be given to the origin of dyspnea.

Kinesiophobia was shown only in studies involving kidney transplant recipients and fatigue [14] and lower level physical activity [15] have been shown to be correlated with kinesiophobia. In the current study it was firstly shown that, 53.5% of patients with CKD at stage 3–4 reported kinesiophobia, which is quite high. Additionally, kinesiophobia was correlated with MEP and fatigue. Interventions about improving respiratory muscle strength may lead to reduce kinesiophobia and fatigue. Therefore, factors that may affect kinesiophobia should be investigated.

Physical activity was adversely affected in patients with CKD. The adverse effects of CKD on peripheral muscle function and chronic inflammation are thought to cause diminished exercise capacity in CKD [40]. In the current study, physical activity level was lower in patients with CKD than in controls. Additionally, 58.1% of patients were inactive, 32.6% were minimally active, and only 9.3% were sufficiently active. It is known that physical activity improves physical functioning; [30] therefore, patients should be directed to physical activity counseling.

The present study has some limitations. Exercise capacity was evaluated using the 6-MWT, a valid and reliable test to evaluate functional exercise capacity [17]. Due to technical problems, no cardiopulmonary exercise test was conducted, but it should be used in future studies. Secondly, physical activity was assessed using a questionnaire. Although the IPAQ is a practical, standardized, and cost-effective assessment tool [30], accelerometers should be used in future studies. Balance was assessed using FRT and BBS, which are standardized tests [25, 26]. However, computer-based systems are recommended for evaluating balance.

## Conclusion

To best our knowledge, this is the first study showed a reduction in proximal upper extremity muscle strength, respiratory muscle strength and an increase in kinesiophobia in patients with CKD stage 3–4. In addition, exercise capacity, lower extremity muscle strength, and physical activity level were lower, dyspnea level was higher, and pulmonary function and balance were lower in patients with CKD at stage 3–4. Furthermore, weight was negatively associated with balance and expiratory muscle strength and fatigue were inversely related to kinesiophobia. Management of CKD is very necessary to prevent adverse CKD-associated outcomes. Additionally, it was emphasized that the treatment strategy should take a holistic approach, addressing comprehensive and coordinated care for various health problems in patients with CKD [41]. Therefore, a detailed assessment including with a wide range of physical and psychological impacts should be done. New studies are needed to find ways to improve outcomes for patients with CKD. Patients with CKD should be directed to rehabilitation programs as early as possible. Cardiopulmonary rehabilitation programs should include exercise training, inspiratory muscle training, and physical activity counseling.

## Data Availability

The datasets used and/or analysed during the current study available from the corresponding author on reasonable request.
